# Supplementary material for: Functional, Immunogenetic, and Structural Convergence in Influenza Immunity between Humans and Macaques
Source: bioRxiv. 2025 Feb 27:2025.02.21.639368. Preprint. [Version 1] doi: 10.1101/2025.02.21.639368 (PMC12190764; doi:10.1101/2025.02.21.639368)
Supplement: Supplement 1 [file NIHPP2025.02.21.639368v1-supplement-1.pdf]

bioRxiv preprint doi: https://doi.org/10.1101/2025.02.21.639368; this version posted February 27, 2025. The copyright holder for this preprint (which was not certified by peer review) is the author/funder. This article is a US Government work. It is not subject to copyright under 17 USC 105 and is also made available for use under a CC0 license.

Supplemental information

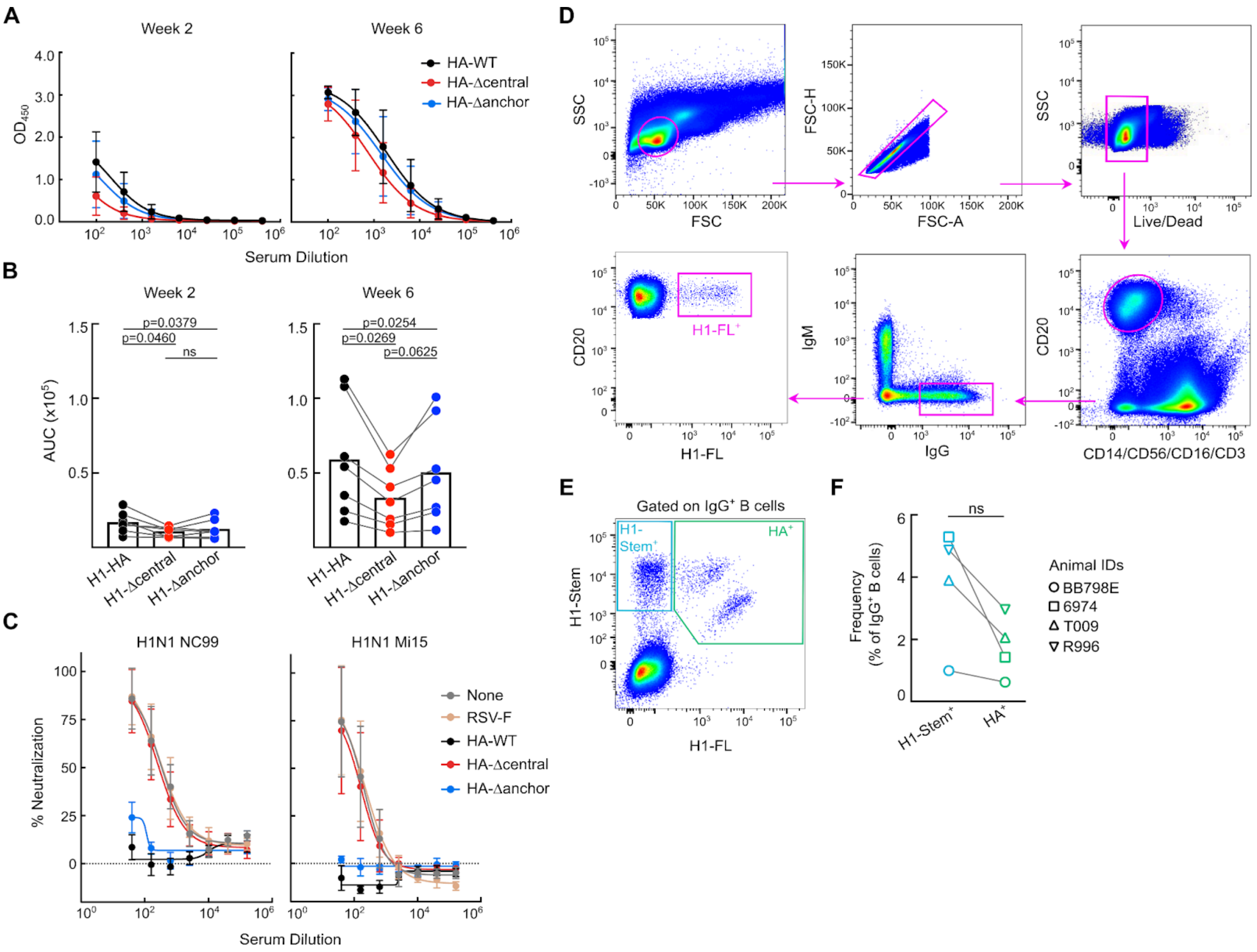

Figure S1. Detailed characteristics of macaque immune responses, related to Figures 1 and 2.

(A-B) Serum reactivity to HA-WT, HA-Δcentral and HA-Δanchor soluble probes at weeks 2 and week 6. Shown are mean and SD (A). Corresponding AUC at weeks 2 and 6. Statistical significance determined by one-way ANOVA with Geisser-Greenhouse correction and Tukeys post hoc test (B).

(C) Serum neutralization curves following serum pre-absorption with RSV-F, HA-WT, HA-Δcentral and HA-Δanchor proteins. Neutralization was measured against H1N1 NC99 and H1N1 MI15 reporter viruses.

(D) Gating strategy to identify antigen specific HA-FL$^+$ B cells. PBMCs were gated on CD3$^-$/CD14$^-$/CD56$^-$/CD16$^-$/CD20$^+$/IgG$^+$/IgM$^-$/H1-FL$^+$.

(E-F) Representative gating strategy to delineate H1-stem single positive and HA+ B cells (gated on IgG$^+$ B cells) (E). Quantification of H1-stem$^+$ (light blue) and HA$^+$ (green) B cells. Significance determined by two tailed Wilcoxon matched-pairs signed rank test (F).

bioRxiv preprint doi: https://doi.org/10.1101/2025.02.21.639368; this version posted February 27, 2025. The copyright holder for this preprint (which was not certified by peer review) is the author/funder. This article is a US Government work. It is not subject to copyright under 17 USC 105 and is also made available for use under a CC0 license.

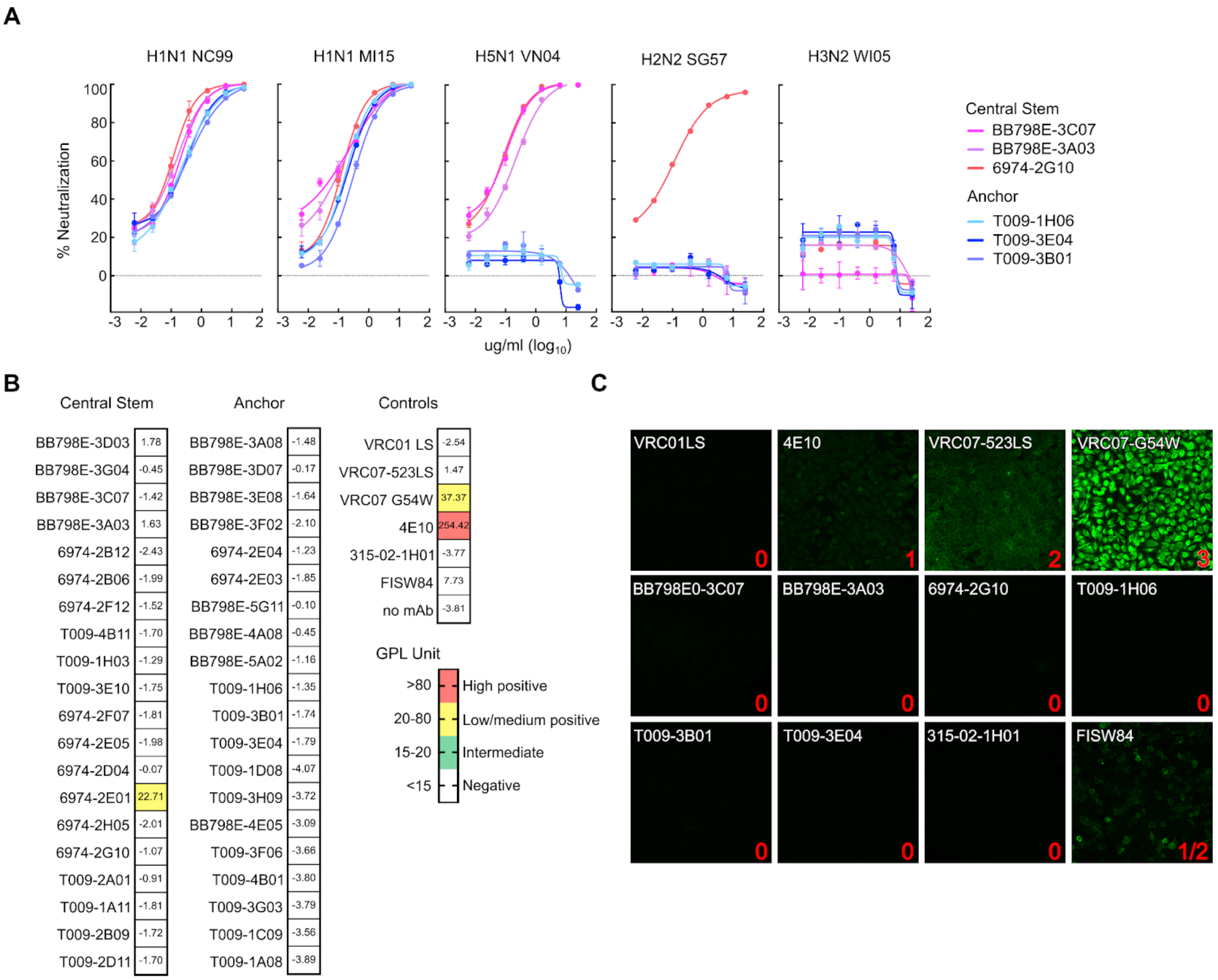

Figure S2. Neutralization and polyreactivity of H1ssF elicited monoclonal antibodies, related to Figure 3.

(A) Neutralization dilution curves for mAbs BB798E-3C07, BB798E-3A03, 6974-2G10, T009-1H06, T009-3E04, T009-3B01 against H1N1 NC99, H1N1 MI15, H5N1 VN04, H2N2 SG57 and H3N2 WI05 reporter viruses. Shown are mean and SD.

(B) IgG phospholipid (GPL) unit value for central stem, anchor epitope and control monoclonal antibodies. GPL score < 20 was considered as not reactive, 20–80 as low positive and > 80 as high positive.

(C) ANA HEp-2 staining of select candidate mAbs: BB798E-3C07, BB798E-3A03, 6974-2G10, T009-1H06, T009-3B01, T009-3E04, compared to control mAbs: 315-02-1H01, FISW84, VRC01LS, 4E10, VRC07-523LS, and VRC07 G54W. Binding to Hep2 cells is scored and indicated (numbered 0 - 3; score 1/2 indicates reactivity between 4E10 and VRC07-523LS).

bioRxiv preprint doi: https://doi.org/10.1101/2025.02.21.639368; this version posted February 27, 2025. The copyright holder for this preprint (which was not certified by peer review) is the author/funder. This article is a US Government work. It is not subject to copyright under 17 USC 105 and is also made available for use under a CC0 license.

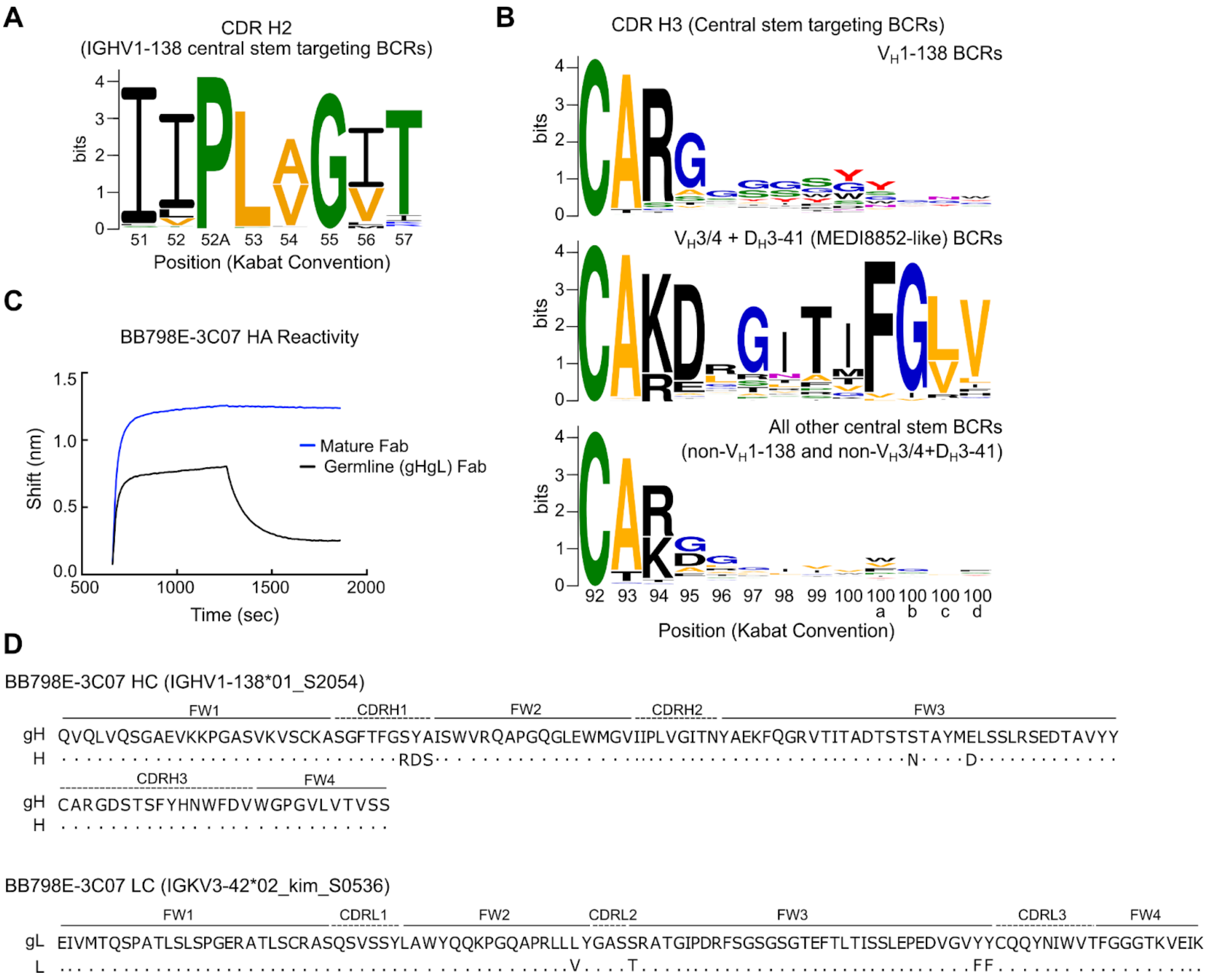

Figure S3. IGHV1-138 central stem mAb characterization, related to Figure 4.

(A) CDR H2 sequence motif of V_H1-138 central stem targeting BCRs from 4 macaques. Position indicated by Kabat convention.

(B) CDR H3 sequence motifs of central stem targeting lineages including V_H1-138 BCRs (top), V_H3/4 + D_H3-41 (MEDI8852-like, middle), and all other central stem targeting BCRs (bottom) from 4 macaques. Tyrosines are colored in red.

(C) BLI sensogram of gHgL (germline) and mature BB798E-3C07 Fab binding to H1 NC99 HA.

(D) Amino acid sequence alignment of mature heavy (H) and light (L) chains and germline reverted heavy (gH) and light (gL) chains of BB798E-3C07.

bioRxiv preprint doi: https://doi.org/10.1101/2025.02.21.639368; this version posted February 27, 2025. The copyright holder for this preprint (which was not certified by peer review) is the author/funder. This article is a US Government work. It is not subject to copyright under 17 USC 105 and is also made available for use under a CC0 license.

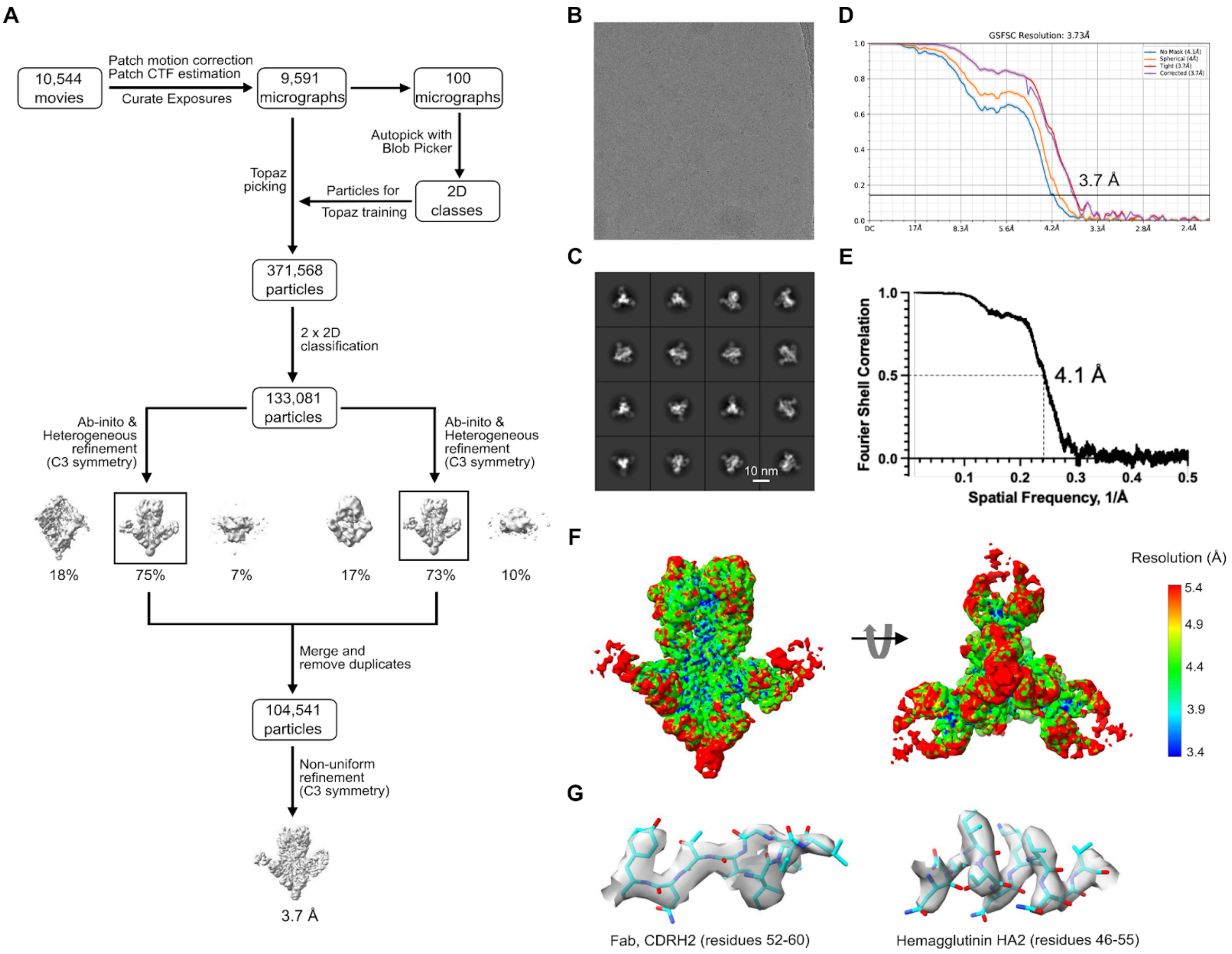

Figure S4. BB798E-3C07 Cryo-EM data processing and workflow, related to Figure 4.

(A) Cryo-EM data processing workflow for HA NC99 in complex with Fab BB798E 3C07.

(B-C) Representative micrographs of 3C07 (B), and representative high-resolution 2D class averages for 3C07 (C).

(D) Gold-standard resolution data generated by cryoSPARC. At the 0.143 threshold, the resolution is 3.7 Å for 3E04.

(E) Fourier shell correlation curve between the map and the atomic model for 3C07.

(F) Results of local resolution analysis using ResMap. The cryo-EM map is colored according to local resolution for 3C07.

(G) Examples of cryo-EM density for 3C07.

bioRxiv preprint doi: https://doi.org/10.1101/2025.02.21.639368; this version posted February 27, 2025. The copyright holder for this preprint (which was not certified by peer review) is the author/funder. This article is a US Government work. It is not subject to copyright under 17 USC 105 and is also made available for use under a CC0 license.

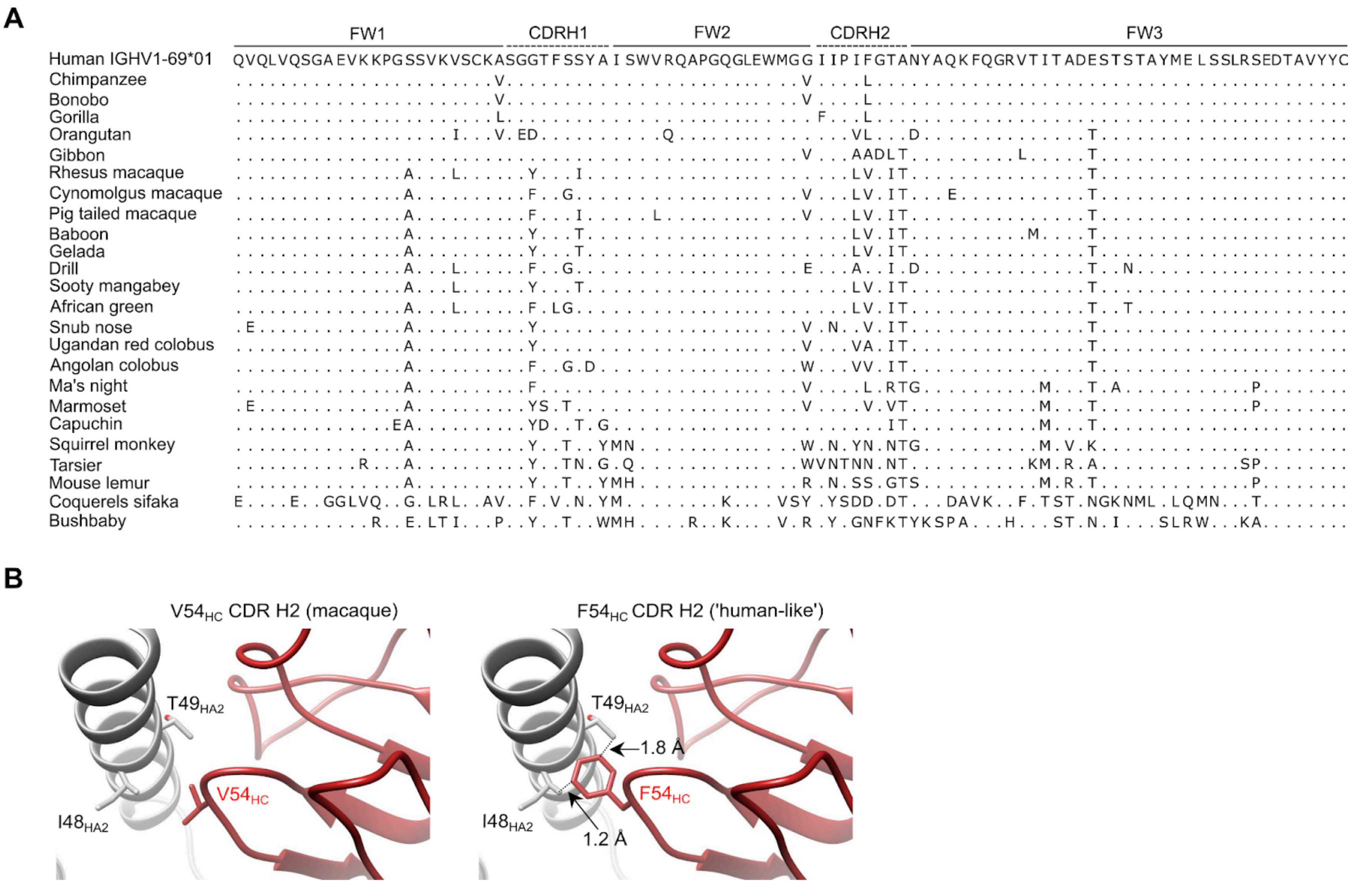

Figure S5. IGHV1-138 V_H- gene characteristics across the primate order, related to Figure 5.

(A) Amino acid alignment of germline human IGHV1-69*01V_H-gene against non-human primate homologs.

(B) Illustration of BB798E-3C07 interaction with HA highlighting the macaque CDR H2 $V54_{HC}$ residue (Left), and a model showing insertion of CDR H2 $F54_{HC}$ and steric clash to $I48_{HA2}$ and $I49_{HA2}$ (Right).

bioRxiv preprint doi: https://doi.org/10.1101/2025.02.21.639368; this version posted February 27, 2025. The copyright holder for this preprint (which was not certified by peer review) is the author/funder. This article is a US Government work. It is not subject to copyright under 17 USC 105 and is also made available for use under a CC0 license.

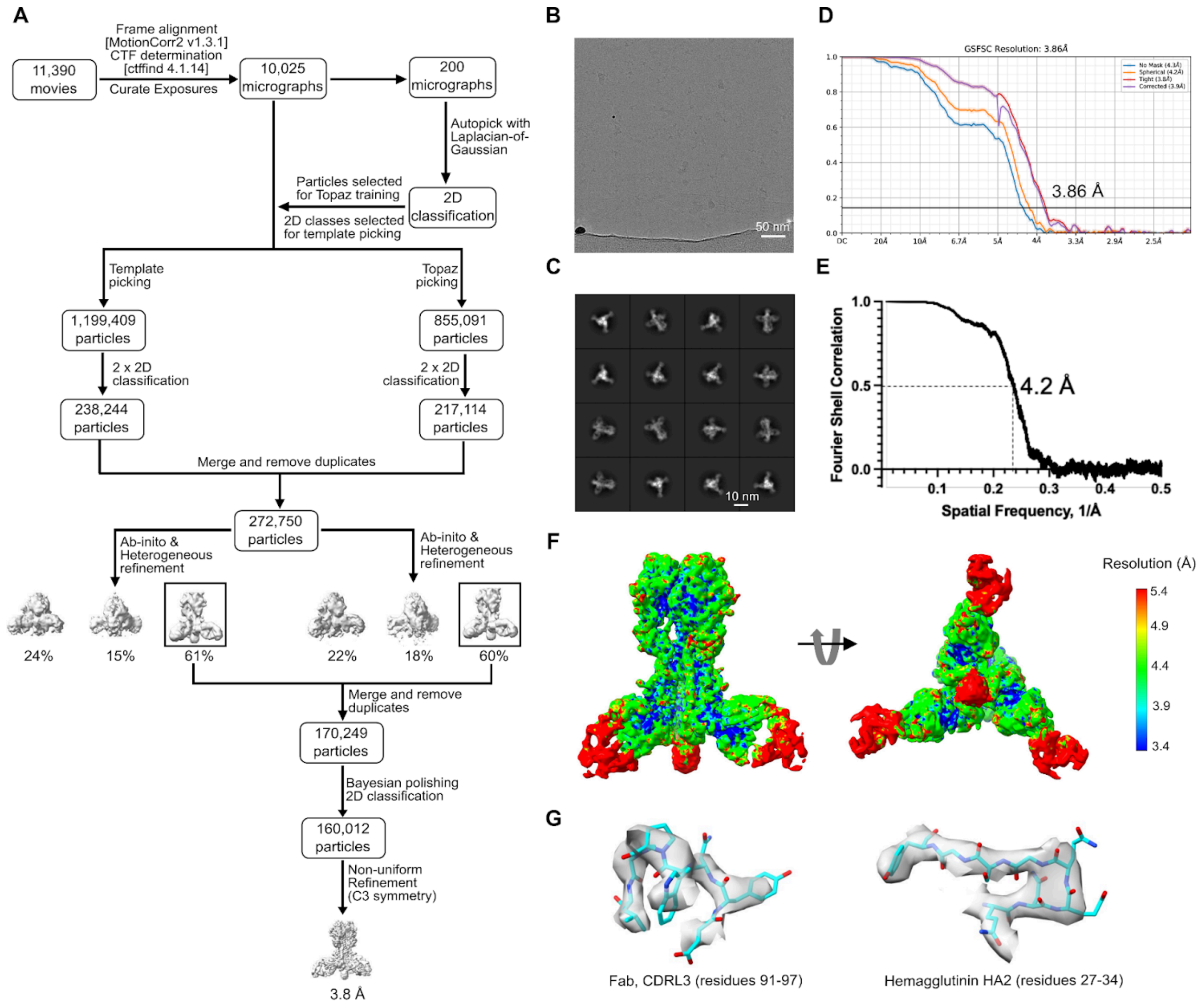

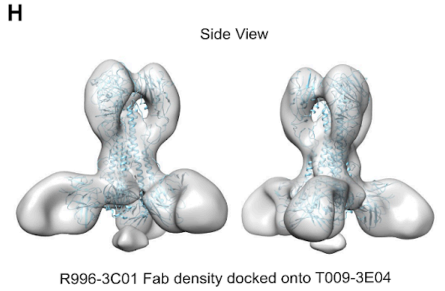

I

Heavy Chain

mAb ID	V_H Gene	Human Ortholog	CDR H2 (aa 51 - 59)	CDR H3 (aa 92 - 103)
907-1E02	IGHV3-48	N/A	ISPTGLSSYY	CARAAKSAIHFNGYDVW
R996-3C01	IGHV3-NL_11*01_S7825	IGHV3-23	ITYTGGSTYY	CAKPALAGIAAPAYFDYW

Light Chain

mAb ID	V_L Gene	Human Ortholog	J_L Gene	CDR L3 (aa 88 - 97)
907-1E02	IGKV3-15	N/A	J_K5	CQQYNSWPPLT
R996-3C01	IGKV3-26-1*01_S2029	IGKV3D-15	J_K4	CQQDYSWPPLT

Figure S6. T009-3E04 Cryo-EM data processing and workflow, related to Figure 6.

(A) Cryo-EM data processing and workflow for HA NC99 in complex with Fab T009 3E04.

(B-C) Representative micrographs of 3E04 (B) and representative high-resolution 2D class averages of 3E04 (C).

(D) Gold-standard resolution data generated by cryoSPARC. At the 0.143 threshold, the resolution is 3.86 Å for 3E04.

bioRxiv preprint doi: https://doi.org/10.1101/2025.02.21.639368; this version posted February 27, 2025. The copyright holder for this preprint (which was not certified by peer review) is the author/funder. This article is a US Government work. It is not subject to copyright under 17 USC 105 and is also made available for use under a CC0 license.

(E) Fourier shell correlation curve between the map and the atomic model for 3E04.

(F) Results of local resolution analysis using ResMap. The cryo-EM map is colored according to local resolution 3E04.

(G) Examples of cryo-EM density for 3E04.

(H) nsEM 3D reconstruction of nsEM R996-3C01 (SWP) Fab docked onto the cryo-EM structure of T009-3E04 (NWP)

(I) HC and LC sequence characteristics of CDR L3 SWP mAbs from human (907-1E02) and macaque (R996-3C01). Sequence characteristics for macaque R996-3C01 are also shown in Figure 5A**.**

bioRxiv preprint doi: https://doi.org/10.1101/2025.02.21.639368; this version posted February 27, 2025. The copyright holder for this preprint (which was not certified by peer review) is the author/funder. This article is a US Government work. It is not subject to copyright under 17 USC 105 and is also made available for use under a CC0 license.

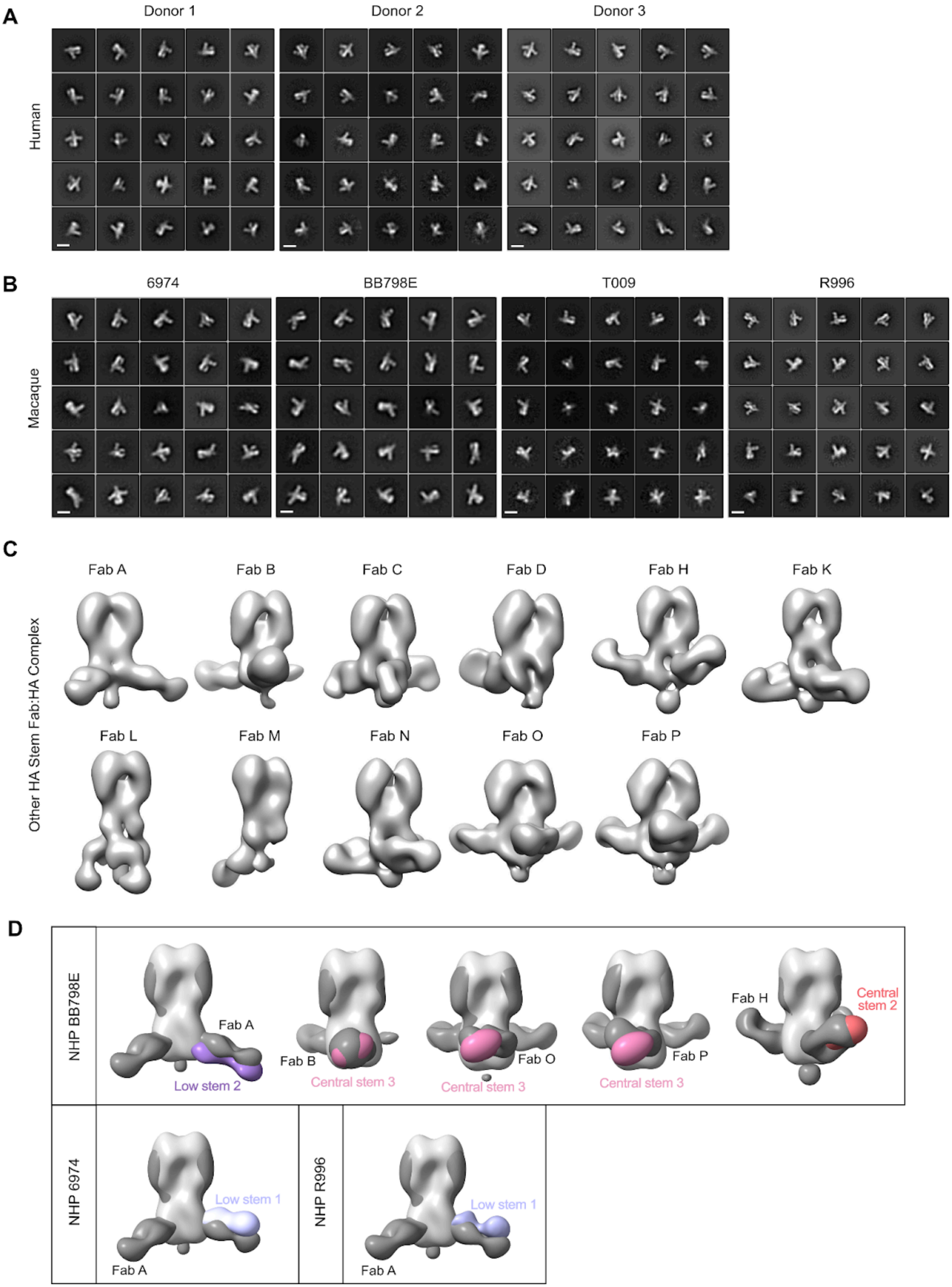

bioRxiv preprint doi: https://doi.org/10.1101/2025.02.21.639368; this version posted February 27, 2025. The copyright holder for this preprint (which was not certified by peer review) is the author/funder. This article is a US Government work. It is not subject to copyright under 17 USC 105 and is also made available for use under a CC0 license.

Figure S7. nsEMPEM polyclonal serum characterization, related to Figure 7.

(A-B) nsEMPEM 2D class averages for each human (A) or macaques (B) serum antibody response. All 2D classification datasets are shown in order of descending particle count. Scale bar is 100Å.

(C) nsEM and 3D reconstruction of other HA-stem macaque Fabs (see Figure 2F) in complex with H1 NC99 HA.

(D) Overlay of other HA-stem macaque Fabs with relevant macaque nsEMPEM density (see Figure 7 C-D).

bioRxiv preprint doi: https://doi.org/10.1101/2025.02.21.639368; this version posted February 27, 2025. The copyright holder for this preprint (which was not certified by peer review) is the author/funder. This article is a US Government work. It is not subject to copyright under 17 USC 105 and is also made available for use under a CC0 license.

Table S1. Cryo-EM data collection and refinement statistics, Related to Figure 4 and 6.

	HA NC99/ Fab BB79E 3-C07	HA NC99/ Fab T009 3-E04
**Data collection and processing**		
Magnification	22,500	22,500
Voltage (kV)	300	300
Electron exposure ($e^{-}/Å^2$)	40	40
Defocus range (μm)	-0.9 to -2.3	-1.0 to -2.7
Pixel size (Å)	1.11	1.11
Symmetry imposed	C3	C3
Initial particle images (no.)	371,568	2,054,500
Final particle images (no.)	104,541	160,012
	EMD-45636 PDB 9CJY	EMD-45637 PDB 9CJZ
Map resolution (Å)	3.73	3.86
FSC threshold	0.143	0.143
Map resolution range (Å)	3.4-5.4	3.4-5.4
**Refinement**		
Initial model used (PDB code)	8D21	8D21
Model resolution (Å)	4.1	4.2
FSC threshold	0.5	0.5
Map sharpening *B* factor ($Å^2$)	-140.5	-158.8
Model composition		
Non-hydrogen atoms	16318	15438
Protein residues	2050	1957
Ligands	0	0
Water	0	0
B factors ($Å^2$)(mean)		
Protein	41.98	168.01
Ligand	N/A	N/A
Water	N/A	N/A
R.m.s. deviations		
Bond lengths (Å)	0.005	0.003
Bond angles (°)	0.597	0.510
Validation		
MolProbity score	1.66	2.01
Clash score	5.34	10.59
Poor rotamers (%)	0.73	0.29
Ramachandran plot		
Favored (%)	94.49	92.54
Allowed (%)	5.51	7.46
Disallowed (%)	0	0
